# Molecular spectrum of KRAS, NRAS, BRAF and PIK3CA mutations in Chinese colorectal cancer patients: analysis of 1,110 cases

**DOI:** 10.1038/srep18678

**Published:** 2015-12-22

**Authors:** Jing Zhang, Jianming Zheng, Yinghong Yang, Junliang Lu, Jie Gao, Tao Lu, Jian Sun, Hui Jiang, Yan Zhu, Yuhui Zheng, Zhiyong Liang, Tonghua Liu

**Affiliations:** 1 Department of Pathology, Peking Union Medical College Hospital, Chinese Academy of Medical Science, Beijing, People’s Republic of China; 2 Department of Pathology, Changhai Hospital of Shanghai, Shanghai, People’s Republic of China; 3 Department of Pathology, Fujian Medical University Union Hospital, Fuzhou, People’s Republic of China

## Abstract

Mutations in genes such as KRAS, NRAS, BRAF and PIK3CA have become an important part of colorectal carcinoma evaluation. The aim of this study was to screen for mutations in these genes in Chinese patients with colorectal cancer (CRC) and to explore their correlations with certain clinicopathological parameters. We tested mutations in the KRAS (exons 2, 3 and 4), NRAS (exons 2, 3 and 4), PIK3CA (exon 20) and BRAF (exon 15) genes using reverse transcriptase-polymerase chain reaction (RT-PCR) and Sanger sequencing in a large cohort of 1,110 Chinese CRC patients who underwent surgical resection at one of three major teaching hospitals located in different regions of China. The prevalence rates of KRAS, NRAS, BRAF and PIK3CA mutations were 45.4%, 3.9%, 3.1% and 3.5%, respectively. Mutant KRAS was associated with the mucinous subtype and greater differentiation, while mutant BRAF was associated with right-sided tumors and poorer differentiation. Our results revealed differences in the genetic profiles of KRAS, NRAS, PIK3CA and BRAF at mutation hotspots between Chinese CRC patients and those of Western countries, while some of these gene features were shared among patients from other Asian countries.

Colorectal cancer (CRC) is the third most common malignancy worldwide. In recent years, the morbidity and mortality due to CRC have risen in the Chinese population. In 2010, the crude incidence rate of CRC in China was 20.90/100,000, and the crude mortality rate was 10.1/100,000, ranking 6th among all cancer sites^1^. Although surgery remains the only curative method for patients with localized tumors, several combinations of chemotherapeutic drugs are used to extend overall and disease-free survival for those with advanced disease^2^.

The development of CRC is a multistep process that results from the accumulation of several genetic alterations. The activation of multiple signaling pathways, specifically RAS-RAF-MAPK and PI3K-PTEN-AKT, plays an important role in regulating cell proliferation, angiogenesis, cell motility, and apoptosis^3^,^4^. Accordingly, the accumulation of mutations in tumor suppressor genes and proto-oncogenes participating in these signaling pathways, such as KRAS, NRAS, BRAF and PIK3CA, significantly contributes to the development of CRC^5^,^6^.

In the treatment of metastatic colorectal cancer, monoclonal antibodies against epidermal growth factor receptor (EGFR), such as cetuximab and panitumumab, have been used in clinical practice since 2004. Approximately 30% to 45% of CRC tumors harbor a KRAS mutation, and mutant KRAS is associated with resistance to anti-EGFR antibodies^7^. While wild-type KRAS appears to be a prerequisite for the response to treatment, it does not necessarily predict the response to anti-EGFR monoclonal antibodies^7^,^8^, indicating that additional genetic alterations might contribute to this non-responsiveness. In addition to KRAS mutations, mutations in other downstream effectors of the EGFR signaling pathway, such as BRAF, NRAS, and components of the PI3K signaling pathway, potentially exert negative effects on the response to anti-EGFR antibodies^9^,^10^,^11^,^12^,^13^,^14^.

To date, numerous investigations into the mutational status of components in the EGFR-RAS-RAF pathway and the PI3K pathway have been conducted and have revealed a diverse distributional pattern of mutations in these genes. However, inconsistency in the prevalence of certain mutations reported in these studies elicits the need for a multicenter study in China in an even larger sample.

In the present study, we aimed to evaluate KRAS, NRAS, BRAF and PIK3CA mutations using both reverse transcriptase-polymerase chain reaction (RT-PCR) and Sanger sequencing in 1,110 samples from Chinese patients with CRC and to determine the frequencies of these mutations and the relationships between these mutations and clinicopathological parameters.

## Materials and Methods

### Samples

The records of all patients diagnosed with CRC from January 2012 to December 2014 at the Department of Pathology, Peking Union Medical College Hospital, Chinese Academy of Medical Sciences, (Beijing, China; 514 cases), the Department of Pathology, Changhai Hospital of Shanghai, Second Military Medical University (Shanghai, China; 299 cases), and the Department of Pathology, Fujian Medical University Union Hospital (Fujian, China; 297 cases) were retrieved. The following exclusion criteria were applied: patients who underwent neoadjuvant therapy before surgery, unavailability of paraffin block specimens for pathology and insufficient clinical information. A total of 1,110 formalin-fixed paraffin-embedded (FFPE) CRC tissue samples were evaluated.

Tumor sections from FFPE tissue samples were stained with hematoxylin–eosin (H&E) and reviewed by two experienced histopathologists independently. Clinicopathological information was obtained by reviewing the medical records in detail and noting the age (≤60 or >60 years), sex (male or female), tumor site (right, left colon or rectum), histological type, differentiation, depth of invasion, lymph node metastasis, distant metastasis and TNM stage. This study was conducted with the approval of the Ethics Committee of all three hospitals, and informed consent was obtained from all patients. The methods were carried out in accordance with the approved guidelines.

### DNA extraction

Sections (5 μm thick) were cut from paraffin-embedded tumor tissue blocks and stained with H&E for histopathological examination. Each sample was evaluated by two experienced pathologists. To obtain maximal tumor DNA, we chose tumor-rich paraffin block specimens whose tumor components were greater than 70% and the amount of stroma was less than 30%. For DNA isolation, 5-μm-thick sections were used for each case. The H&E section was used as a reference, and tumor-rich regions of the sections were trimmed off from the slides based on their respective H&E staining patterns and transferred to lysate buffer (included in the DNA purification kit). DNA in the collected tissue samples was extracted using the QIAGEN QIAamp DNA FFPE Tissue Kit (Cat No. 56404, Qiagen, Shanghai, China) following the manufacturer’s protocol. DNA from each sample was eluted in 50 μl of ATE buffer (included in the kit).

### Amplification-refractory mutation system-polymerase chain reaction (ARMS-PCR) for KRAS/NRAS/PIK3CA/BRAF mutations

We tested mutations in the KRAS (exons 2, 3 and 4), NRAS (exons 2, 3 and 4), PIK3CA (exon 20) and BRAF (exon 15) genes (mutations detectable with the AmoyDx Kit are detailed in Supplementary Table 1) using the Chinese Food and Drug Administration (CFDA)-approved AmoyDx Human KRAS/NRAS/PIK3CA/BRAF Mutation Detection Kit (Amoy Diagnostics Co. Ltd, Xiamen, China). The quality of the extracted DNA was evaluated by amplifying a housekeeping gene and using the HEX channel provided with the kit. Amplifications were performed for 47 cycles (95 °C for 5 min, 1 cycle; 95 °C for 25 s, 64 °C for 20 s, and 72 °C for 20 s, 15 cycles; and 93 °C for 25 s, 60 °C for 35 s, and 72 °C for 20 s, 31 cycles). FAM and HEX signals were collected during the third stage. Run files were analyzed and interpreted as specified in the manufacturer’s manual.

### Sanger sequencing

Targeted exons of the selected genes, including KRAS (exons 2, 3 and 4), NRAS (exons 2, 3 and 4), PIK3CA (exon 20) and BRAF (exon 15), were first amplified by PCR. Amplicons were then sequenced with an ABI 3730XL sequencer (Life Technologies, Carlsbad, CA, USA). Detailed information about primers, cycling conditions and buffers are presented in Supplementary Table 2.

### Next-generation sequencing (NGS)

In cases where the results of ARMS-PCR and Sanger sequencing diverged, the particular specimens were further analyzed using an Ion torrent PGM sequencer with the Ion AmpliSeq Colon and Lung Cancer Panel (Life Technologies, USA). NGS results were perceived as the gold standard in the present study. However, for cases in which the NGS assays failed, the ARMS-PCR results were used in statistical analyses, taking into account that the ARMS-PCR kit is CFDA-approved and presumably has a higher sensitivity and specificity than Sanger sequencing.

### Statistical analysis

The data were processed using the SPSS 17.0 statistical software (SPSS, Inc., Chicago, IL, USA). Individual information and baseline characteristics were summarized using descriptive statistics. The chi-square (χ^2^) test or, where appropriate, the Fisher’s exact test was used to compare the proportion of gene mutations among groups with different clinicopathological factors. An independent sample t test was adopted to compare the age of the patients with different genetic mutations. Multivariate logistic regression analysis was performed to investigate the effects of covariates on gene mutations, using a backward stepwise (likelihood ratio) method with the odds ratio (OR) calculated. The two-sided significance level was set at 0.05.

## Results

### Clinicopathological characteristics of CRC patients

In this study, 1,110 FFPE tissue blocks were retrieved. The prevalence of CRC was higher in males (58.6%, 649/1,110) than in females (41.4%, 461/1,110). The median age of the patient cohort was 62.1 years, ranging from 18 to 96 years. Regarding the histological subtypes, 92.3% (1024/1,110) of tumors were tubular adenocarcinomas, and 7.7% (86/1,110) were mucinous adenocarcinomas. The tumors were graded according to the WHO criteria (WHO Classification of Tumours of the Digestive System, Fourth Edition) as follows: 211 (19.0%) were well differentiated, 816 (73.5%) were moderately differentiated and 83 (7.5%) were poorly differentiated. The locations of the primary tumors included the rectum, the left side of the colon (including sigmoid colon and splenic flexure) and the right side of the colon (cecum, hepatic flexure, transverse colon). In total, 314 (28.3%) samples were located in the left colon, 249 (22.4%) were located in the right colon, and 547 (49.3%) were located in the rectum.

### Consistency among ARMS-PCR, Sanger sequencing and NGS results

ARMS-PCR was successful in all 1,110 cases, while Sanger sequencing failed in 13 cases. Detailed nucleotide changes detected by Sanger sequencing are listed in Supplementary Table 3. Inconsistencies between ARMS-PCR and Sanger sequencing occurred in 4.9% (54/1,110) of cases. In 20.4% (11/54) of the inconsistent cases, two mutations were detected by the ARMS-PCR assay, but only one of them was detected by Sanger sequencing. In the remaining 79.6% (43/54) of cases, the ARMS-PCR assay detected one mutation, while Sanger sequencing detected no mutations. Further validation with NGS sided with ARMS-PCR in 63.0% (34/54) of the inconsistent cases. A total of 9.3% (5/54) of the cases showed two mutations that were detected by ARMS-PCR, while only one of the mutations was detected by NGS. A total of 16.7% (9/54) of the cases showed one mutation that was detected by ARMS-PCR but were deemed mutation-negative by NGS. A total of 11.1% (6/54) of the cases failed to yield sufficient DNA for NGS analysis.

### Distribution of KRAS, NRAS, PIK3CA and BRAF mutations in colorectal carcinomas

The distribution of KRAS, NRAS, PIK3CA and BRAF mutations in the 1,110 Chinese CRC patient samples is presented in Table 1. KRAS mutations were detected in 45.4% (504/1,110) of the cases. In this study, 40.0% (443/1,110) of KRAS mutations were in exon 2, 1.4% (16/1,110) were in exon 3, and 4.1% (45/1,110) were in exon 4. Within KRAS exon 2, 79.0% of the mutations were detected in codon 12, and 21.0% were identified in codon 13. The most prevalent mutation was G12D, which accounted for 40.7% of all of the exon 2 mutations, followed by G13D and G12V (20.5% each). Eight tumors showed two KRAS mutations.

In total, 3.9% (43/1,110) of the samples harbored an NRAS mutation: 2.2% (24/1,110) in exon 2 (codon 12 or 13), 1.7% (19/1,110) in exon 3 (codon 61), and none in exon 4 (codon 146). Eight tumors showed both KRAS and NRAS mutations.

The presence of PIK3CA mutations was noted in 39 cases (3.5%, 39/1,110). Twenty-six tumors had both KRAS and PIK3CA mutations. PIK3CA mutations were present in 26 of 504 (5.2%) patients with KRAS mutations, compared with only 13 of 606 (2.1%) patients with wild-type KRAS (Supplementary Table 4). This finding suggests that PIK3CA and KRAS gene mutations represent partially overlapping subgroups in colon cancer.

BRAF exon 15 mutations were detected in 34 of the 1,110 CRC patients (3.1%). The mutual exclusivity of KRAS (exons 2, 3 and 4), NRAS (exons 2, 3 and 4) and BRAF mutations was confirmed, given that none of the patients with a KRAS or NRAS mutation harbored a simultaneous BRAF mutation. However, three tumors showed concurrent mutations in BRAF and PIK3CA.

### KRAS, NRAS, PIK3CA and BRAF mutations and their correlations with clinicopathological findings

A summary of the relationships among KRAS, NRAS, PIK3CA and BRAF mutations and various clinicopathological features is provided in Table 2. KRAS mutations were significantly more prevalent in tumors in the right side of the colon than in tumors in the left side or the rectum (53.4% vs. 35.0% vs. 47.7%, respectively; P < 0.001). Meanwhile, KRAS mutations were significantly associated with well and moderately differentiated tumors but less so with poorly differentiated tumors (48.3% vs. 46.1% vs. 31.3%, respectively; P = 0.023). KRAS mutations were also more prevalent in mucinous than tubular adenocarcinomas (61.6% vs. 44.0%, respectively; P = 0.002). However, no significant relationship was observed between KRAS mutations and sex (P = 0.288), age (P = 0.185), depth of invasion (P = 0.694), lymph node metastasis (P = 0.410), distant metastasis (P = 0.103) or TNM stage (P = 0.209).

Similar to KRAS mutations, BRAF mutations occurred more frequently in right-sided tumors (8.4%, 21/249) than those located in the left side (1.9%, 6/314) or the rectum (1.3%, 7/547) (P < 0.001). Unlike KRAS mutations, BRAF mutations were more common in poorly differentiated tumors (13.3%, 11/83) than in well and moderately differentiated tumors (0.9% vs. 2.6%) (P < 0.001).

Associations between PIK3CA or NRAS mutations and patient characteristics, such as age, gender, tumor location, histological type, tumor differentiation, invasion depth, lymph node metastasis, distant metastasis or TNM stage, were statistically insignificant.

In the multivariate logistic regression analyses, mutant KRAS was associated with the mucinous subtype (P = 0.001) and greater differentiation (P = 0.027), while mutant BRAF was associated with right-sided tumors (P < 0.001) and poorer differentiation (P < 0.001) (Table 3).

## Discussion

EGFR signaling plays a key role in the development and progression of CRC. In particular, this receptor triggers downstream signaling cascades, including the RAS-RAF-MAPK and PI3K-AKT pathways, to stimulate cell proliferation, differentiation, survival and invasion^15^,^16^. Recently, significant improvements have been made in the treatment of metastatic CRC, with the aid of monoclonal antibodies targeting EGFR, such as cetuximab and panitumumab^17^,^18^. However, these anti-EGFR therapies have been effective only in a subset of CRC patients^19^. KRAS mutations negatively predict the response to EGFR-targeted therapies in metastatic CRC patients^20^,^21^. However, only 40% to 60% of KRAS wild-type patients respond to treatment; thus, a considerable number of patients do not benefit from anti-EGFR therapy^18^. It is also important to identify other important molecular alterations that may impact anti-EGFR therapy. De Roock *et al.* studied the effect of other EGFR downstream mutations on the efficacy of cetuximab in the largest cohort of patients with chemotherapy-refractory metastatic CRC treated with cetuximab plus chemotherapy^12^. Their findings not only confirmed the negative effect of KRAS mutations on the outcome after cetuximab but also demonstrated that BRAF, NRAS, and PIK3CA exon 20 mutations were associated with a low response rate^12^. Gene mutations in the EGFR signaling pathway, such as mutations in KRAS, NRAS, BRAF and PIK3CA, have become an important part of CRC evaluation, and their alterations may determine the therapeutic response to anti-EGFR therapy^12^,^13^.

In the present study, we evaluated KRAS, NRAS, BRAF, and PIK3CA somatic mutation frequencies in a large cohort of 1,110 CRC patients from three major teaching hospitals located in different regions of China. The present study was a large retrospective, multicenter study that utilized three methods to analyze the mutational profile of Chinese CRC patients. Additionally, we assessed the correlations of these genetic mutations with clinicopathological features. The prevalence rates of KRAS, NRAS, BRAF, and PIK3CA somatic mutations were 45.4%, 3.9%, 3.5%, and 3.1%, respectively. In the remaining 44.1% of patients, no mutations were detected in any of the targets analyzed. These results suggest that approximately 10% of CRC patients harbor NRAS, BRAF, or PIK3CA mutations in a KRAS wild-type population.

The frequency of KRAS mutations varies worldwide, ranging from 13% to 66%^22^,^23^,^24^,^25^,^26^,^27^,^28^,^29^,^30^,^31^,^32^,^33^,^34^,^35^,^36^,^37^,^38^. The frequency of KRAS mutations (45.4%) in the current study was consistent with published data from Asian countries (i.e., China, Japan, and India) (20–66%) and Western countries (i.e., USA, France, and Germany) (13–53%). The wide variability of the results of different studies may be attributed to the ethnicity, geographical distribution, and techniques used in previous studies^31^,^33^,^34^,^36^.

In our study, KRAS mutations in codons 12, 13, 61, 117 and 146 were evaluated. The majority of the mutations occurred in codon 12 or 13 (87.9%). The KRAS G12D mutation was the most common, followed by the G12V, G13D, G12S, G12C, G12A, G12R, and G13C mutations. These data differ slightly from the studies of Western populations, suggesting that race might play a role in KRAS mutation patterns^39^.

Our findings suggest that tumors with KRAS mutations are significantly more associated with well- and moderately differentiated tumors compared to poorly differentiated tumors, which is consistent with a previous report^13^. In addition, KRAS mutations occurred more frequently in tumors on the right side of the colon compared to those on the left side and in the rectum. Interestingly, tumors with a KRAS mutation tended to occur more frequently in mucinous adenocarcinomas compared with the tubular subtype, which further supports previous findings. Other clinicopathological features, such as sex, age, depth of invasion, lymph node metastasis or TNM stage, did not exhibit any association with KRAS mutations, which is consistent with a recent report of Chinese patients^22^.

BRAF is a member of the RAF gene family and acts as a downstream effector of activated KRAS. The frequency of BRAF mutations varies widely from 1.1% to 25% worldwide^22^,^23^,^24^,^25^,^26^,^27^,^28^,^29^,^30^,^31^,^32^,^33^,^34^,^35^,^36^,^37^,^38^. The V600E mutation frequency of 3.1% observed in this study is consistent with various Asian studies (1.1% to 5.8%) but is lower than several Western studies^33^,^34^,^36^. This difference in the mutation frequency may be attributable to different sample selections, ethnicities and geographical distributions. Notably, BRAF mutations were more common on the right side of the colon than on the left side of the colon and the rectum, which is similar to the findings of a previous study^36^. Furthermore, none of the BRAF-mutated samples harbored a concurrent KRAS mutation, indicating that these mutations are mutually exclusive, which is consistent with previous findings^29^,^40^ (Supplementary Table 4). BRAF mutations were also more common in poorly differentiated tumors than well and moderately differentiated tumors, which supports previous findings^13^.

Notably, in several Western population-based studies, mutant BRAF was either strongly associated with clinicopathological characteristics such as older age and female gender or at least showed a trend toward such associations, while this was not the case in the present study^41^,^42^,^43^. In our study, the age and gender distribution were identical in BRAF mutant and BRAF wild-type patients. This finding is interesting because in another large Chinese cohort study by Shen *et al.* such an association was neither established nor was a trend shown^13^. Thus, we conclude that such a discrepancy might not be the result of limited BRAF mutant cases in the sample, but rather a distinct difference between Western and Chinese populations. BRAF associations in western populations might be secondary to other primary unknown associations such as life style (diet, tobacco…) that are probably different between western and Chinese populations. However, a more in-depth investigation is needed to further elucidate the underlying mechanisms.

The PIK3CA gene encodes the p110 catalytic subunit of PI3K that regulates this signaling pathway^3^. Prognostic and treatment-predictive effects have not been firmly established. De Roock *et al.* reported that PIK3CA exon 20 mutations were significantly associated with a low response rate^12^. A recent meta-analysis also reported that PIK3CA exon 20 mutations were associated with reduced response rates and OS^44^. We detected PIK3CA exon 20 mutations because the role of PIK3CA exon 9 mutations was not clear. PIK3CA mutations occur in 2% to 18% of metastatic CRCs; in this study, the frequency of PIK3CA mutation was 3.5%, which is consistent with other Asian studies^24^,^25^,^26^,^27^,^28^,^29^. PIK3CA mutations also often coexist with KRAS mutations^22^,^25^,^29^,^30^,^45^.

We found a significant association between PIK3CA and KRAS mutation (P = 0.007). However, no significant associations between PIK3CA mutations and clinical characteristics were observed in the present study, which is consistent with earlier studies^22^,^29^ (Supplementary Table 4).

As one of the RAS family members, NRAS contains effector binding domains identical to KRAS. Thus, NRAS mutations in codons 12, 13, 61 and 146 yield effects similar to those observed as a result of KRAS activation^46^. Unlike KRAS mutations, which constitute a large percentage of the mutations in colorectal cancer, NRAS mutations are rare. To date, there are few data available concerning the prevalence of NRAS mutations. Irahara *et al.* reported a mutation incidence of 2.2%^47^, whereas a 4.19% mutation rate was reported in a Chinese study^13^, and a rate of 9.57% was reported in Greek and Romanian patients^48^. We detected NRAS mutations in 3.9% of the tumors in the present study. This discrepancy may be attributed to different diagnostic techniques, ethnicities, genetic factors, and geographical distributions. NRAS mutations can coexist with KRAS mutations, but no significant association between these mutations was noted in this study. Similarly, no significant associations between NRAS mutations and clinical characteristics were observed in our study (Supplementary Table 4).

Discrepancies in the prevalence of several mutations might raise concern. However, it is agreed upon that the development and progression of CRC is a multi-step process that involves the acquisition of numerous mutations. Thus, the geographical distribution of patients, which results in different physical, biochemical and environmental exposures to the colon, could have a profound impact on the somatic mutations of the tumor. The present study enrolled patients from 3 major teaching hospitals located in different regions (north, east and southeast) of China to optimize the geographical representativeness of CRC patients. Second, as the accumulation of mutations may be associated with more advanced stages of colorectal cancer, the proportions of advanced diseases in the sample might interfere with the prevalence of mutations. The present study enrolled consecutive, non-selected cases but was still biased by the fact that most of the patients had operable disease upon admission. In other words, most of the patients in the present study were absent for distant metastasis, thus representing the profile of patients with earlier disease. The above considerations might help to explain the lower mutation prevalence in the present study in comparison with other studies.

The present study suffered from the following limitations. First, as stated above, patient selection bias could not be completely eliminated despite our best efforts. Thus, our results should be interpreted with the understanding that our samples represented cases of earlier-stage disease in the Chinese population. Second, due to how recently our patients were diagnosed, follow-up information such as distant metastasis, recurrence, and therapeutic response was incomplete. Third, some hotspot mutations such as in exon 11 of the BRAF gene and in exon 9 of the PIK3CA gene were not screened due to the limitation of the ARMS-PCR kit. Additionally, genetic alterations on the mRNA and protein levels, as well as epigenetic deregulation of CRC, were not discussed. A study that enrolls more stage IV CRC patients at multiple institutions should be incorporated into the present study along with follow-up information to provide a clearer insight into targeted therapy for CRC patients in China. An in-depth investigation into other genetic deregulations in CRC may also aid in the identification of novel targets for personalized therapy in the future.

## Conclusions

In conclusion, our results revealed differences in the genetic profiles of KRAS, NRAS, PIK3CA and BRAF at mutation hotspots between Chinese CRC patients and those from Western countries, while some of these gene features were shared among patients from other Asian countries.

## Additional Information

**How to cite this article**: Zhang, J. *et al.* Molecular spectrum of KRAS, NRAS, BRAF and PIK3CA mutations in Chinese colorectal cancer patients: analysis of 1,110 cases. *Sci. Rep.* **5**, 18678; doi: 10.1038/srep18678 (2015).

## Supplementary Material

Supplementary Information

## Figures and Tables

**Table 1:** Types of RAS/BRAF/PIK3CA mutations detected in 1,110 cases of Chinese CRC.

Gene	Exon	Codon	Mutation	Numbers of mutations (% of 1, 110)
KRAS	2	12, 13	G12S, G12D, G12C, G12R, G12V, G12A, G13C, G13D,	443 (40.0)
KRAS	3	61	Q61L, Q61R, Q61H	16 (1.4)
KRAS	4	117, 146	K117N, A146T, A146V, A146P	45 (4.1)
NRAS	2	12, 13	G12D, G12S, G13R, G12C, G12V, G12A, G13V,	24 (2.2)
NRAS	3	61	Q61R, Q61K, Q61L, Q61H	19 (1.7)
NRAS	4	146	A146T	0 (0)
BRAF	15	600	V600E, V600K, V600R, V600D	34 (3.1)
PIK3CA	20	1047	H1047R, H1047L	39 (3.5)

**Table 2:** Correlation between KRAS, NRAS, BRAF and PIK3CA mutations and clinicopathological parameters in CRC.

		Total number N = 1, 110 (%)	KRAS status			NRAS status			BRAF status			PIK3CA status		
Clinicopathological features			Mutation n (%)	Wild-type n (%)	P value	Mutation n (%)	Wild-type n (%)	P value	Mutation n (%)	Wild-type n (%)	P value	Mutation n (%)	Wild-type n (%)	P value
Gender	Male	649 (58.6)	286 (44.1)	363 (55.9)	0.288	25 (3.9)	624 (96.1)	0.964	18 (2.8)	631 (97.2)	0.507	22 (3.4)	627 (96.6)	0.791
	Female	461 (41.4)	218 (47.3)	243 (52.7)		18 (3.9)	443 (96.1)		16 (3.5)	445 (96.5)		17 (3.7)	444 (96.3)	
Age (years)	Mean ± SD	62 (18–96)	62.10 ± 12.2	62.12 ± 12.2	0.185	62.28 ± 13.9	61.54 ± 12.1	0.695	61.35 ± 11.0	61.57 ± 12.2	0.918	60.97 ± 11.3	61.59 ± 12.2	0.758
Tumor site	Right	249 (22.4)	133 (53.4)	116 (46.6)	<0.001	9 (3.6)	240 (96.4)	0.659	21 (8.4)	228 (91.6)	<0.001	14 (5.6)	235 (94.4)	0.115
	Left	314 (28.3)	110 (35.0)	204 (65.0)		10 (3.2)	304 (96.8)		6 (1.9)	308 (98.1)		10 (3.2)	304 (96.8)	
	Rectum	547 (49.3)	261 (47.7)	286 (52.3)		24 (4.4)	523 (95.6)		7 (1.3)	540 (98.7)		15 (2.7)	532 (97.3)	
Histological type	Tubular	1024 (92.3)	451 (44.0)	573 (56.0)	0.002	42 (4.1)	982 (95.9)	0.175	29 (2.8)	995 (97.2)	0.123	38 (3.7)	986 (96.3)	0.357
	Mucinous	86 (7.7)	53 (61.6)	33 (38.4)		1 (1.2)	85 (98.8)		5 (5.8)	81 (94.2)		1 (1.2)	85 (98.8)	
Differentiation	Well	211 (19.0)	102 (48.3)	109 (51.7)	0.023	10 (4.7)	201 (95.3)	0.633	2 (0.9)	209 (99.1)	<0.001	13 (6.2)	198 (93.8)	0.063
	Moderate	816 (73.5)	376 (46.1)	440 (53.9)		31 (3.8)	785 (96.2)		21 (2.6)	795 (97.4)		23 (2.8)	793 (97.2)	
	Poor	83 (7.5)	26 (31.3)	57 (68.7)		2 (2.4)	81 (97.6)		11 (13.3)	72 (86.7)		3 (3.6)	80 (96.4)	
Depth of invasion	T1	24 (2.2)	11 (45.8)	13 (54.2)	0.694	3 (12.5)	21 (87.5)	0.065	1 (4.2)	23 (95.8)	0.074	0 (0)	24 (100.0)	0.512
	T2	188 (16.9)	90 (47.9)	98 (52.1)		4 (2.1)	184 (97.9)		6 (3.2)	182 (96.8)		9 (4.8)	179 (95.2)	
	T3	841 (75.8)	374 (44.5)	467 (55.5)		35 (4.2)	806 (95.8)		22 (2.6)	819 (97.4)		29 (3.4)	812 (96.6)	
	T4	57 (5.1)	29 (50.9)	28 (49.1)		1 (1.8)	56 (98.2)		5 (8.8)	52 (91.2)		1 (1.8)	56 (98.2)	
Lymph node metastasis	Yes	485 (43.7)	227 (46.8)	258 (53.2)	0.410	20 (4.1)	465 (95.9)	0.704	19 (3.9)	466 (96.1)	0.135	14 (2.9)	471 (97.1)	0.318
	No	625 (56.3)	277 (44.3)	348 (55.7)		23 (3.7)	602 (96.3)		15 (2.4)	610 (97.6)		25 (4.0)	600 (96.0)	
Distant metastasis	Yes	32 (2.9)	10 (31.3)	22 (68.7)	0.103	3 (9.4)	29 (90.6)	0.123	4 (12.5)	28 (87.5)	0.009	3 (9.4)	29 (90.6)	0.099
	No	1078 (97.1)	494 (45.8)	584 (54.2)		40 (3.7)	1038 (96.3)		30 (2.8)	1048 (97.2)		36 (3.3)	1042 (96.7)	
TNM stage	I	159 (14.3)	77 (48.4)	82 (51.6)	0.209	4 (2.5)	155 (97.5)	0.329	7 (4.4)	152 (95.6)	0.019	7 (4.4)	152 (95.6)	0.153
	II	469 (42.3)	204 (43.5)	265 (56.5)		19 (4.2)	450 (95.8)		8 (1.7)	461 (98.3)		18 (3.8)	451 (96.2)	
	III	450 (40.5)	213 (47.3)	237 (52.7)		17 (3.8)	433 (96.2)		15 (3.3)	435 (96.7)		11 (2.4)	439 (97.6)	
	IV	32 (2.9)	10 (31.3)	22 (68.7)		3 (9.4)	29 (90.6)		4 (12.5)	28 (87.5)		3 (9.4)	29 (90.6)	

**Table 3:** Multivariate logistic regression analysis of the relationship between gene mutations and clinicopathological characteristics in CRC patient.

Clinicopathological features	KRAS status			NRAS status			BRAF status			PIK3CA status		
	OR	95% CI	P	OR	95% CI	P	OR	95% CI	P	OR	95% CI	P
Gender	0.864	0.677–1.104	0.242	1.011	0.54–1.894	0.972	0.826	0.399–1.709	0.606	0.964	0.502–1.851	0.912
Age (>60 or not)	1.214	0.952–1.547	0.118	1.156	0.619–2.160	0.650	1.142	0.534–2.439	0.733	1.024	0.531–1.971	0.945
Tumor site	0.977	0.837–1.141	0.767	1.136	0.749–1.722	0.548	0.375	0.232–0.604	<0.001	0.688	0.459–1.031	0.070
Histological type	2.219	1.396–3.526	0.001	0.274	0.037–2.050	0.207	1.476	0.517–4.213	0.467	0.262	0.034–2.023	0.199
Differentiation	0.759	0.594–0.969	0.027	0.715	0.389–1.314	0.280	4.063	1.975–8.356	<0.001	0.563	0.304–1.043	0.068
Depth of invasion	1.117	0.812–1.537	0.495	0.557	0.273–1.136	0.107	1.444	0.561–3.713	0.446	1.234	0.477–3.191	0.664
Lymph node metastasis	1.766	0.991–3.147	0.054	0.340	0.079–1.470	0.149	5.980	1.424–25.107	0.015	1.145	0.256–5.120	0.859
Distant metastasis	0.699	0.283–1.723	0.436	1.408	0.24–8.243	0.705	7.657	1.443–40.64	0.017	7.634	1.244–46.843	0.028
TNM stage	0.696	0.439–1.103	0.123	2.815	0.891–8.893	0.078	0.242	0.076–0.777	0.017	0.631	0.186–2.142	0.460

OR: odds ratio; 95% CI: 95% confidence interval.
