# Supplementary material for: Molecular spectrum of KRAS, NRAS, BRAF and PIK3CA mutations in Chinese colorectal cancer patients: analysis of 1,110 cases
Source: Sci Rep. 2015 Dec 22;5:18678. doi: 10.1038/srep18678 (PMC4687048; doi:10.1038/srep18678)
Supplement: Supplementary Information [file srep18678-s1.doc]

**Molecular spectrum of KRAS, NRAS, BRAF and PIK3CA mutations in Chinese colorectal cancer patients: analysis of 1110 cases**

Jing Zhang, Jianming Zheng, YinghongYang, Junliang Lu, Jie Gao, Tao Lu, Jian Sun, Hui Jiang, Yan Zhu, Yuhui Zheng, Zhiyong Liang, Tonghua Liu

**Supplementary Tables**

**Supplementary Table** 1. Targeted mutations of AmoyDx ARMS-PCR kit.

| **Gene** | **Well*** | **Exon** | **Aminoacid change** | **Base change** | **Cosmic ID** |
| --- | --- | --- | --- | --- | --- |
| **KRAS** | 1 | 2 | G12S | 34G>A | 517 |
|  |  |  | G12D | 35G>A | 521 |
|  | 2 | 2 | G12C | 34G>T | 516 |
|  |  |  | G12R | 34G>C | 518 |
|  |  |  | G12V | 35G>T | 520 |
|  |  |  | G12A | 35G>C | 522 |
|  |  |  | G13C | 37G>T | 527 |
|  | 3 | 2 | G13D | 38G>A | 532 |
|  | 4 | 3 | Q61L | 182A>T | 553 |
|  |  |  | Q61R | 182A>G | 552 |
|  |  |  | Q61H | 183A>C | 554 |
|  |  |  | Q61H | 183A>T | 555 |
|  | 5 | 4 | K117N | 351A>C | 19940 |
|  |  |  | K117N | 351A>T | 28519 |
|  |  |  | A146T | 436G>A | 19404 |
|  |  |  | A146V | 437C>T | 19900 |
|  |  |  | A146P | 436G>C | 19905 |
| **NRAS** | 6 | 2 | G12D | 35G>A | 564 |
|  |  |  | G12S | 34G>A | 563 |
|  | 7 | 3 | G13R | 37G>C | 569 |
|  |  |  | G12C | 34G>T | 562 |
|  |  |  | G12V | 35G>T | 566 |
|  |  |  | G12A | 35G>C | 565 |
|  |  |  | G13V | 38G>T | 574 |
|  | 8 | 3 | Q61R | 182A>G | 584 |
|  |  |  | Q61K | 181C>A | 580 |
|  |  |  | Q61L | 182A>T | 583 |
|  |  |  | Q61H | 183A>C | 586 |
|  | 9 | 4 | A146T | 436G>A | 27174 |
| **PIK3CA** | 10 | 20 | H1047R | 3140A>G | 775 |
|  |  |  | H1047L | 3140A>T | 775 |
| **BRAF** | 11 | 15 | V600E1 | 1799T>A | 476 |
|  |  |  | V600K | 1798_1799GT>AA(Complex) | 473 |
|  |  |  | V600E2 | 1799_1800TG>AA(Complex) | 475 |
|  |  |  | V600R | 1798_1799GT>AG(Complex) | 474 |
|  |  |  | V600D1 | 1799_1800TG>AC(Complex) | \ |
|  |  |  | V600D2 | 1799_1800TG>AT(Complex) | 477 |

*An amplification curve in a well represents one of the mutations listed behind the well number. The AmoyDx ARMS-PCR kit is incapable of distinguishing specific mutations in one well.

**Supplementary Table 2. Detailed information for Sanger Sequencing.**

a.Primers for Sanger Sequencing.

| Target Region | Primer | Sequence | Length  （bp） |
| --- | --- | --- | --- |
| *KRAS*  exon2 | K-M3-F25 | GTTCTAATATAGTCACATTTTCA | 202 |
|  | KRAS-R6 | TGGTCCTGCACCAGTAATATG |  |
| *KRAS*  exon3 | K-Q61-seq-F1 | GAGCGGATAACAATTTCACACAGG | 364 |
|  | K-Q61-seq-R1 | CGCCAGGGTTTTCCCAGTCACGAC |  |
| *KRAS*  exon4 | K-EX4-ce-F1 | ATGACAAAAGTTGTGGACAGGTTTTGA | 248 |
|  | K-EX4-ce-R1 | TGTTACTTACCTGTCTTGTCTTTGCTGATG |  |
| *NRAS*  exon2 | NRAS-M6-F2 | TACTGGTTTCCAACAGGTTCTTGCT | 160 |
|  | NRAS-M3-R1 | CTACCACTGGGCCTCACCTCTAT |  |
| *NRAS*  exon3 | NRAS-M1-F1 | CCAGGATTCTTACAGAAAA | 166 |
|  | NRAS-M2-R2 | CGCAAATGACTTGCTATTATTGATGGC |  |
| *NRAS*  exon4 | NRAS-EX4-ce-F1 | GCCACTGTACCCAGCCTAATCTTG | 283 |
|  | NRAS -M12-R1 | CACATCTCTACCAGAGTTAATCAACTGATGC |  |
| *PIK3CA*  exon20 | PI-ce-F2 | CAGGAGATGTGTTACAA | 255 |
|  | PI-ce-R2 | ATGCTGTTTAATTGTGTGGAAGATC |  |
| *BRAF*  exon15 | B-ce-F | GCTCTGATAGGAAAAT | 235 |
|  | B-ce-R | TAGTAACTCAGCAGCATCT |  |

b. Buffering and cycling conditions for KRAS and NRAS.

| Solution | Volume（µL） | Cycling |
| --- | --- | --- |
| H_2_O | 15.5 | 95℃(5min)  95℃(25s)  56-63℃(30s) 50 cycles  72℃(20s)  72℃(10min)  Annealing temperature was 56℃for *KRAS*exon2，63℃ for*NRAS*exon 4,62℃ for other targets。 |
| 10*TaKaRa Buffer（containing Mg^2+^） | 2.5 |
| 1# Mg^2+^ | 3.5 |
| 12# dNTP+U | 0.2 |
| Primer (Forward) | 0.05 |
| Primer (Reverse) | 0.05 |
| HS-Taq（5U/µL） | 0.2 |
| Template DNA | 3 |
| Total Volume | 25 |

c)Buffering and cycling conditions for PIK3CA.

| Solution | Volume（µL） | Cycling |
| --- | --- | --- |
| H_2_O | 32 | 95℃(5min)  95℃(25s)  58℃(30s) 50 Cycles  72℃ (20s)  72℃ (10min) |
| 10*TaKaRa Buffer（containing Mg^2+^） | 5 |
| 1# Mg^2+^ | 7 |
| 12# dNTP+U | 0.4 |
| PI-ce-F2 | 0.1 |
| PI-ce-R2 | 0.1 |
| HS-Taq（5U/µL） | 0.4 |
| Template DNA | 5 |
| Total Volume | 50 |

d)Buffering and cycling conditions for BRAF.

| Solution | Volume（µL） | Cycling |
| --- | --- | --- |
| H_2_O | 32.4 | 95℃(5min)  95℃(25s)  60℃(30s) 50 Cycles  72℃ (20s)  72℃ (10min) |
| 10*TaKaRa Buffer（Containing Mg^2+^） | 5 |
| 1# Mg^2+^ | 6 |
| 12# dNTP+U | 0.4 |
| B-ce-F | 0.4 |
| B-ce-R | 0.4 |
| HS-Taq（5U/µL） | 0.4 |
| Template DNA | 5 |
| Total Volume | 50 |

**Supplementary Table 3.Base changes of KRAS, NRAS, PIK3CA and BRAF genes in 1097* cases.**

| **Gene** | **Aminoacid change** | **Base change** | **Count** | **Prevalence** |
| --- | --- | --- | --- | --- |
| **KRAS** | G12D | 35G>A | 171 | 15.59% |
|  | G12S | 34G>A | 33 | 3.01% |
|  | G12A | 35G>C | 15 | 1.37% |
|  | G12V | 35G>T | 86 | 7.84% |
|  | G12R | 34G>C | 3 | 0.27% |
|  | G12C | 34G>T | 24 | 2.19% |
|  | G13C | 37G>T | 2 | 0.18% |
|  | G13D | 38G>A | 86 | 7.84% |
|  | A59T | 175G>A | 3 | 0.27% |
|  | Q61L | 182A>T | 1 | 0.09% |
|  | Q61R | 182A>G | 1 | 0.09% |
|  | Q61H | 183A>C | 9 | 0.82% |
|  | Q61H | 183A>T | 2 | 0.18% |
|  | K117N | 351A>C | 5 | 0.46% |
|  | K117N | 351A>T | 5 | 0.46% |
|  | A146T | 436G>A | 22 | 2.01% |
|  | A146V | 437C>T | 6 | 0.55% |
|  | A146P | 436G>C | 5 | 0.46% |
| **NRAS** | G12D | 35G>A | 12 | 1.09% |
|  | G12S | 34G>A | 0 | 0.00% |
|  | G13D | 38G>A | 2 | 0.18% |
|  | G13R | 37G>C | 4 | 0.36% |
|  | G12C | 34G>T | 3 | 0.27% |
|  | G12V | 35G>T | 1 | 0.09% |
|  | G12A | 35G>C | 0 | 0.00% |
|  | G13V | 38G>T | 1 | 0.09% |
|  | Q61R | 182A>G | 6 | 0.55% |
|  | Q61K | 181C>A | 8 | 0.73% |
|  | Q61L | 182A>T | 1 | 0.09% |
|  | Q61H | 183A>C | 2 | 0.18% |
| **PIK3CA** | H1047R | 3140A>G | 32 | 2.92% |
|  | H1047L | 3140A>T | 3 | 0.27% |
| **BRAF** | V600E1 | 1799T>A | 33 | 3.01% |

Sanger sequencing failed in 13 cases.

**Supplementary Table 4. Associations between KRAS, NRAS, BRAF and PIK3CA gene mutations.**

| **Genes** |  | **KRAS** | | **p** | **NRAS** | | **P** | **PIK3CA** | | **p** |
| --- | --- | --- | --- | --- | --- | --- | --- | --- | --- | --- |
|  |  | **Mutation** | **Wild** |  | **Mutation** | **Wild** |  | **Mutation** | **Wild** |  |
| **KRAS** | **Mutation** |  |  |  | 8 | 496 | <0.001 | 26 | 478 | 0.005 |
|  | **Wild** |  |  |  | 35 | 571 |  | 13 | 593 |  |
| **NRAS** | **Mutation** | 8 | 35 | <0.001 |  |  |  | 1 | 42 | 0.993 |
|  | **Wild** | 496 | 571 |  |  |  |  | 38 | 1029 |  |
| **PIK3CA** | **Mutation** | 26 | 13 | 0.005 | 1 | 38 | 0.993 |  |  |  |
|  | **Wild** | 478 | 593 |  | 42 | 1029 |  |  |  |  |
| **BRAF** | **Mutation** | 1 | 33 | <0.001 | 0 | 34 | 0.461 | 3 | 31 | 0.217 |
|  | **Wild** | 503 | 573 |  | 43 | 1033 |  | 36 | 1040 |  |
